# Bovine Serum Albumin–Trypsin Sponges for Enhanced Enzymatic Stability and Protein Digestion Efficiency

**DOI:** 10.1021/acsabm.5c01038

**Published:** 2025-09-19

**Authors:** Maria Kaeek, Luai R. Khoury

**Affiliations:** 26747 Technion Israel Institute of Technology, Department of Materials Science and Engineering, Haifa 32000, Israel

**Keywords:** Engineered Protein Scaffolds, Proteolytic Biocatalysis, Enzyme Immobilization, Biocatalytic Sponges, Nanoscale FRET Imaging

## Abstract

Protein-based materials are emerging as versatile platforms for biocatalysis and biomedical applications due to their structural tunability and intrinsic catalytic capabilities. Here, we present a light-activated strategy for the scalable fabrication of enzymatically active sponges via covalent cross-linking of trypsin within a bovine serum albumin (BSA) matrix. This method leverages photoinitiated Tyr–Tyr coupling, creating a nanoscale enzyme distribution that addresses critical limitations observed in conventional enzyme immobilization methods—namely, instability, autolysis, and restricted reusability. By modulating trypsin concentration and acetic acid (AA) during synthesis, we achieve precise control over cross-link density, enhancing both mechanical flexibility and catalytic accessibility. The sponges retain over 50% of their enzymatic activity after 30 days of storage and maintain ∼60% functionality across ten reuse cycles. Structural integrity and enzyme distribution were validated by attenuated total reflection–Fourier transform infrared (ATR–FTIR) and fluorescence resonance energy transfer (FRET) microscopy, revealing preserved secondary structure and uniform spatial embedding. Proteolytic performance was benchmarked against Cytochrome c, Concanavalin A, and Fetal Bovine Serum, demonstrating enhanced cleavage efficiency and substrate accessibility. This light-activated, reusable platform introduces a scalable approach for stable enzyme immobilization with broad implications for proteomics, biocatalysis, therapeutic devices, and advanced biomedical diagnostics.

## Graphical abstract

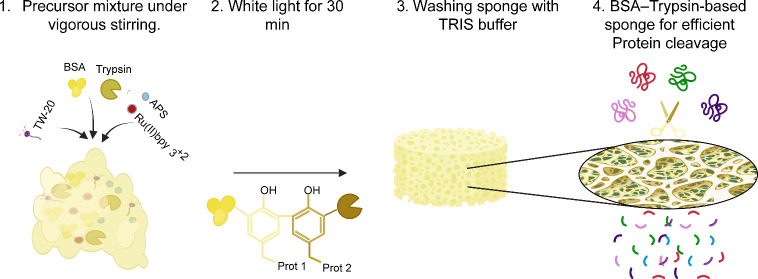

## Introduction

Enzymes, once studied primarily for their roles in metabolic pathways, are now being repurposed as programmable catalysts in materials science, offering selective reactivity under mild, tunable conditions. Their intrinsic substrate specificity and activity across diverse environments - including physiological, acidic, and near-neutral conditions - have expanded their applications well beyond traditional biochemical contexts, finding use in polymer synthesis, hydrogel design, and bioactive interfaces.^1,2^

Among their many functions, enzymatic digestion remains central to biochemistry and proteomics, enabling the systematic breakdown of complex proteins into peptides for downstream analysis and identification.^3,4^ However, despite its widespread utility, enzymatic digestion faces critical limitations. Incomplete cleavage can generate peptides of suboptimal length, reducing sequence coverage and increasing sample complexity.^5^ Additionally, proteins with compact tertiary structures or extensive post-translational modifications can limit enzyme accessibility and compromising digestion efficiency.^6,7^ To overcome these challenges, several strategies have been explored, including enzyme immobilization and the development of engineered or alternative proteases, aimed at improving digestion reproducibility, specificity, and overall proteome coverage.^8,9^

Trypsin, a serine protease widely used in proteomics, cleaves peptide bonds at the carboxyl side of lysine and arginine residues. Its optimal activity under mildly alkaline conditions (pH 7.5–8) and physiological temperature (37 °C) makes it a standard tool for peptide mapping and targeted protein fragmentation in analytical workflows.^10,11^ However, in its free form, trypsin is prone to autolysis, thermal degradation, and rapid loss of catalytic activity during extended digestion procedures, limiting its long-term utility and reproducibility.^12,13^

To overcome the limitations of free trypsin, two complementary strategies have been explored: protein engineering and enzyme immobilization. The former involves modifying trypsin’s primary structure to improve resistance to autolysis and thermal denaturation.^14^ The latter - more broadly applicable - relies on anchoring enzymes to solid matrices, thereby enhancing their structural stability, thermal resilience, and reusability.^15^ Various immobilization platforms have been investigated for trypsin stabilization. Silica-based matrices, for example, protect the enzyme’s tertiary structure and extend its operational lifespan.^16^ In one study, trypsin embedded within a double-network silica–acrylamide hydrogel retained over 80% of its activity after three digestion cycles.^17^ In another, magnetic silica microspheres functionalized with trypsin enabled rapid microwave-assisted digestions, maintaining high activity over seven consecutive cycles.^18^ Biodegradable polymeric sponges have also shown promise for sustained enzyme release, supporting continuous digestion in contexts such as in vivo proteolysis and implantable bioactive systems.^19^ More recently, macroporous hydrogels have emerged as versatile scaffolds for multienzyme immobilization, facilitating sequential catalytic steps and improving enzyme dispersion.^20^

While enzyme immobilization offers clear benefits - including enhanced stability, reusability, and process control - it also presents significant challenges, particularly when applied to sensitive enzymes like trypsin. A primary concern is enzyme deactivation resulting from conformational changes during the immobilization process. Harsh chemical conditions, rigid support matrices, or incompatible cross-linking chemistries can disrupt the enzyme’s active site or impede substrate accessibility, ultimately diminishing catalytic efficiency.^21^ Furthermore, nonspecific binding interactions may lead to enzyme leaching or detachment, especially under flow or mechanical agitation.^22^ In systems where immobilization compromises activity, compensation is often attempted by increasing enzyme loading, an approach that escalates material costs, raises reaction viscosity, and complicates downstream purification.^23,24^

To address the limitations of conventional immobilization, we developed a covalently cross-linked BSA–trypsin sponge via a one-step photochemical process, in which trypsin is directly integrated into the proteinaceous matrix through Tyr–Tyr cross-linking with bovine serum albumin (BSA). Unlike traditional approaches that rely on physical multistep adsorption or surface tethering, this embedded configuration enhances enzymatic stability while minimizing autolysis and leaching. The design builds on our previously established BSA-based sponge platform, which demonstrated high porosity, mechanical flexibility, and compressive resilience.^25^ Here, we extend this platform to create enzymatically active sponges that merge BSA’s structural robustness and tunable porosity with trypsin’s catalytic functionality, resulting in improved operational stability and sustained activity across repeated digestion cycles.

Our fabrication strategy involves generating a protein-based foam by mixing trypsin, BSA, and the surfactant Tween-20 (TW-20) with ammonium persulfate (APS) and Ru­(II) bipyridyl dication (Ru­(bpy)_3_^+2^) as photoinitiated cross-linking agents.^26^ Upon exposure to white light, tyrosine residues on BSA and trypsin undergo covalent Tyr–Tyr coupling, yielding a stable, enzymatically active sponge. To modulate the cross-linking density and enhance catalytic performance, acetic acid (AA) was introduced during synthesis. Varying the AA concentration enabled us to fine-tune the balance between mechanical integrity and enzyme accessibility, thereby enhancing digestion efficiency without requiring high enzyme loading.^27^

We evaluated the structural and functional properties of the resulting sponges using multiple characterization techniques. Attenuated Total Reflection–Fourier Transform Infrared (ATR–FTIR) spectroscopy confirmed the retention of trypsin’s secondary structure within the BSA matrix, while Fluorescence Resonance Energy Transfer (FRET) microscopy revealed effective enzyme incorporation and spatial distribution throughout the sponge. The sponges exhibited robust enzymatic activity over 10 repeated digestion cycles, with minimal performance loss. When stored under refrigeration, they retained approximately 50% of their initial activity after 30 days, demonstrating notable stability.

To assess substrate compatibility, the sponges were tested against structurally diverse proteins, including Cytochrome c, the membrane-associated lectin Concanavalin A, and fetal bovine serum (FBS).^28,29^ Sodium Dodecyl Sulfate–Polyacrylamide Gel Electrophoresis (SDS-PAGE) and liquid chromatography–tandem mass spectrometry (LC–MS/MS) analyses confirmed efficient digestion across these targets, highlighting the sponge’s potential for applications in proteomics and biocatalysis.

## Results

### Optimizing Enzyme Functionality through BSA-Trypsin-Based Sponge Fabrication: An Innovative Strategy for Enhanced Protein Digestion

To fabricate BSA–trypsin hybrid sponges, we employed TW-20 as the primary foaming agent due to its high solubility and foaming capacity.^30,31^ BSA and trypsin were dissolved in 20 mM TW-20 solution at 4 °C to minimize premature proteolytic degradation.^32,33^ The final BSA concentration was fixed at 132 mg/mL (2 mM),^34,35^ while trypsin concentration was systematically varied (0, 0.0015, 0.01, 0.4, and 1 mg/mL) to determine the optimal balance between catalytic activity and structural robustness.

Photoactivated cross-linking was initiated by adding Ru­(bpy)_3_^+2^ and APS at a 15:1:1 volume ratio.^36,37^ The mixture was homogenized at 15,000 rpm for 2.5 min to produce a foam with high porosity and mechanical integrity, as established in our previous work.^25^ The foam was transferred into cylindrical molds (8 mm diameter × 4.5 mm height) and exposed to white light for 30 min at room temperature, facilitating Tyr–Tyr cross-linking between BSA and trypsin.^38,39^ The resulting covalently cross-linked sponges were washed extensively with TRIS to remove residual reagents and prepared for further analysis (Figure 1).

**Figure 1:**
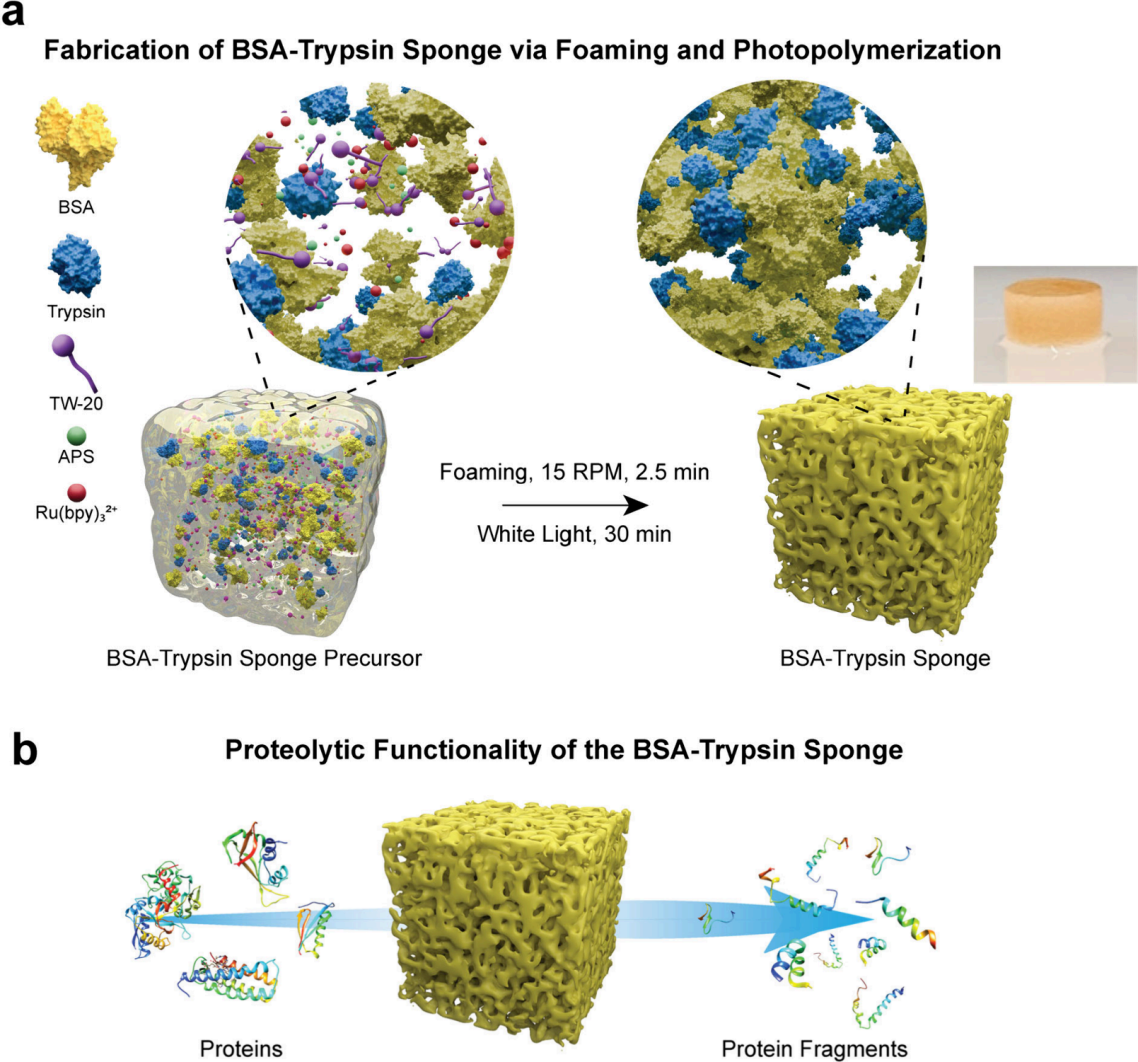
Fabrication of BSA–trypsin sponges and their enzymatic digestion capability. (a) Schematic representation of the one-step synthesis process. BSA, trypsin, Ru­(bpy)_3_^+2^, APS, and the surfactant TW-20 are mixed at 4 °C to form a homogeneous precursor solution. Upon foaming and exposure to white light, photoinduced Tyr–Tyr cross-linking covalently embeds trypsin within the BSA network, generating a porous and enzymatically active sponge. Left inset: A magnified view of the precursor highlights the dispersed proteins and reactive agents prior to cross-linking. Right inset: Upon light activation, a highly interconnected porous network is formed, in which trypsin is covalently bound within the BSA scaffold. The resulting structure contains continuous microchannels and pore walls enriched with enzymatic sites. (b) Functional illustration of protein digestion. Upon contact with target proteins, the enzyme-rich sponge facilitates efficient substrate cleavage, releasing peptide fragments. The controlled network enhances trypsin stability, accessibility, and reusability, supporting sustained proteolysis across multiple digestion cycles.

One of the primary challenges when incorporating enzymes into a solid matrix is maintaining their native structure and functionality. To assess the structural fidelity of trypsin after incorporation into the sponge, we prepared a trypsin-only sponge and analyzed its secondary structure using ATR–FTIR spectroscopy, focusing on the Amide I region (1600–1700 cm^–1^).^40^ Deconvolution of this region enabled quantitative comparison of α-helix, β-sheet, β-turn, and random coil content in both free and embedded trypsin^41,42^ (Figure S1a,b).

The ATR-FTIR spectra revealed distinct peaks within the Amide I region, representing specific secondary structures: β-turns (1680–1660 cm^–1^), α-helices (1660–1649 cm^–1^), random coils (1648–1638 cm^–1^), and β-sheets (1637–1615 cm^–1^).^43,44^ Analysis of trypsin in solution revealed a distribution of 20.4 ± 0.2% α-helix, 46.8 ± 0.2% β-sheet, 18.2 ± 0.3% β-turn, and 14.5 ± 0.4% random coil. In contrast, trypsin in the trypsin-based sponge exhibited a slightly altered composition, with 16.7 ± 2.5% α-helix, 39.4 ± 3.2% β-sheet, 23.5 ± 0.2% β-turn, and 20.4 ± 1.0% random coil (Figure S1c). Although the sponge form showed an increase in β-turns and random coils, the substantial retention of β-sheet content—falling within theoretical ranges for trypsin—indicates preserved structural integrity. This is particularly important because β-sheets play a central role in maintaining the enzyme’s core stability and active site conformation, which are essential for its catalytic functionality.^44,45^

Together, these findings demonstrate that our light-triggered cross-linking approach preserves the native secondary structure of trypsin during sponge fabrication. This structural fidelity is essential for maintaining catalytic activity and underscores a key advantage of our protein–protein cocrosslinking strategy.

### Fluorescence-Based Mapping of Trypsin–BSA Integration via FRET Microscopy

To investigate the spatial distribution and molecular integration of trypsin within the BSA-based sponge matrix, we employed FRET microscopy - a sensitive technique for detecting nanoscale protein proximity.^46,47^ This represents one of the first applications of FRET to visualize protein–protein interactions within porous enzymatic sponges. The noninvasive nature of this approach enabled real-time mapping of molecular proximity, offering insights into enzyme distribution and nanoscale embedding within the sponge framework - both critical for ensuring catalytic functionality and long-term stability.

For FRET imaging, BSA was labeled with fluorescein isothiocyanate (FITC, donor) and trypsin with rhodamine B isothiocyanate (RBITC, acceptor) (Figure 2a). Dual-labeled proteins were used during sponge fabrication to enable direct visualization of their colocalization within the matrix. Confocal imaging revealed strong, spatially uniform fluorescence signals from both labels. Channel 1 (λ_em_ = 484–540 nm) confirmed homogeneous FITC–BSA distribution, forming the sponge’s structural backbone (Figure 2a­(i)). Channel 2 (λ_em_ = 565–797 nm) displayed red fluorescence from RBITC–trypsin, verifying its spatial incorporation into the matrix (Figure 2a­(ii)). FRET signals were detected in Channel 3 (λ_ex_ = 488 nm, λ_em_ = 565–797 nm), confirming close molecular proximity between donor and acceptor fluorophores (Figure 2a­(iii)). Merged images revealed overlapping signals as magenta regions, highlighting widespread colocalization of trypsin with BSA throughout the sponge network (Figure 2a­(iv)).

**Figure 2:**
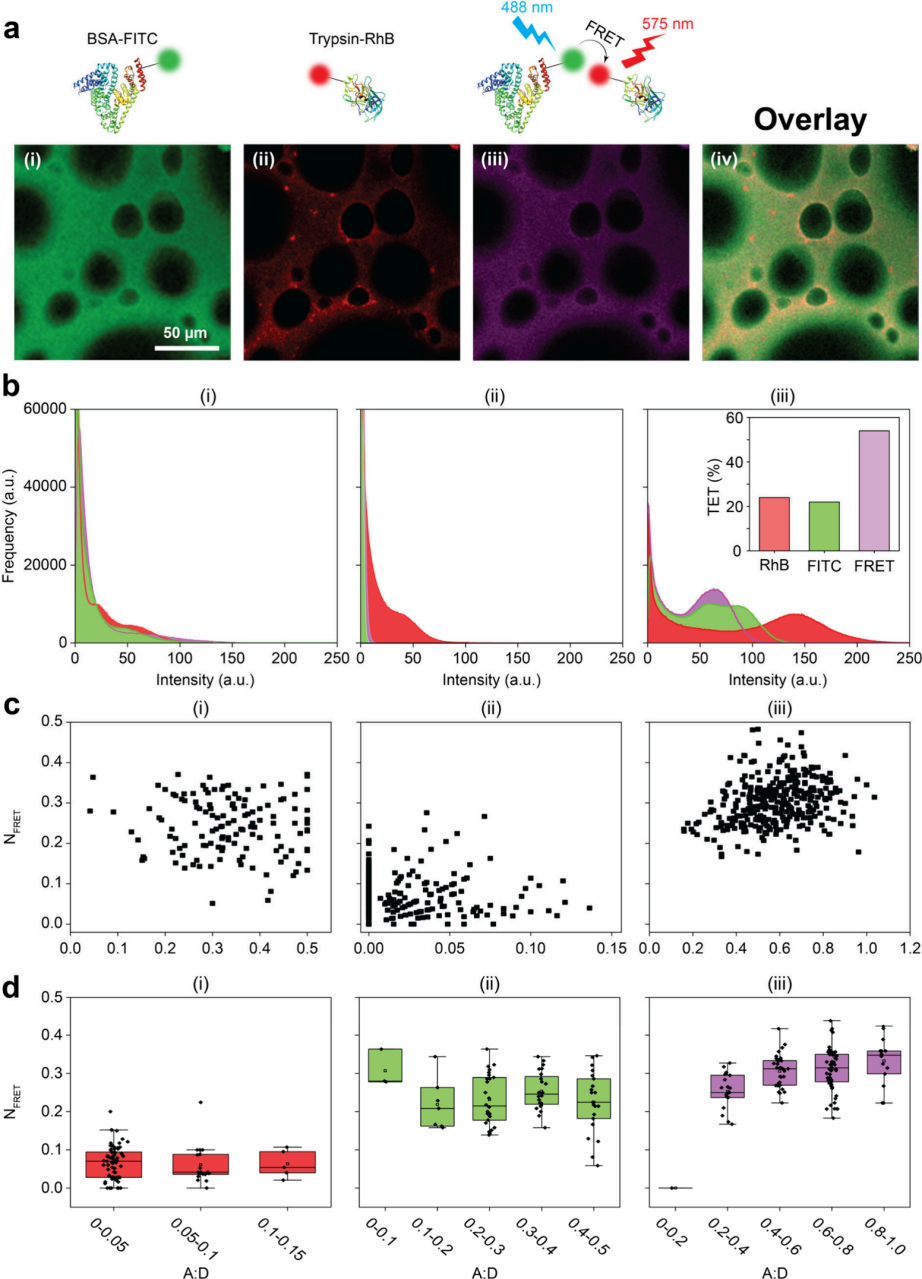
FRET-based visualization and quantification of trypsin–BSA colocalization in cross-linked sponges. (a) Schematic illustration of the FRET mechanism between FITC-labeled BSA and RBITC-labeled trypsin within the covalently cross-linked sponge matrix. Upon FITC excitation, energy is transferred to RBITC when the two proteins are in close spatial proximity, generating a FRET signal that confirms molecular interaction. (i) Green fluorescence signal from FITC-labeled BSA within the sponge matrix. (ii) Red fluorescence signal from RBITC-labeled trypsin within the sponge structure. (iii) FRET signal in purple, indicating regions of close proximity between BSA and trypsin in the sponge. (iv) Overlay of both channels, showing colocalized regions of BSA and trypsin throughout the sponge. (b) Fluorescence intensity histograms for BSA-trypsin-based sponges. (i) FITC and (ii) RBITC channels show the distribution of donor and acceptor signals, respectively. (iii) FRET channel shows strong magenta emission, confirming efficient energy transfer and indicating close spatial proximity between BSA and trypsin within the sponge matrix. Inset: TET efficiency highlights the dominant contribution of FRET over individual fluorophores. (c) Scatter plots of *N*_FRET_ versus *A*:*D* ratios. (i) and (ii) show minimal *N*_FRET_ values in samples labeled with only FITC or only RBITC, respectively. In the absence of both donor and acceptor, no energy transfer occurs. (iii) Dual-labeled sponges exhibit clustered *N*_FRET_ values across a broad *A*:*D* range, indicating effective energy transfer and consistent BSA–trypsin interactions within the matrix. (d) Box plots illustrating *N*_FRET_ distribution for different conditions. (i) Shows consistently low *N*_FRET_ values for samples where only FITC is used, indicating the absence of FRET without an acceptor. (ii) Displays *N*_FRET_ values for samples where only RBITC is used, confirming the lack of FRET due to the absence of the donor. (iii) Shows box plots for samples containing both FITC and RBITC, with increased *N*_FRET_ values correlating with higher *A*:*D* ratios, indicating efficient energy transfer and confirming the close spatial proximity between BSA and trypsin within the sponge matrix.

To quantitatively assess fluorescence intensity and spatial distribution, histograms were generated for each channel. Channel 1 and 2 histograms represented FITC and RBITC intensity profiles, reflecting the distribution of BSA and trypsin, respectively (Figure 2b­(i–ii)). The FRET channel (Figure 2b­(iii)) revealed a significant magenta signal, consistent with energy transfer and donor–acceptor proximity, while the quantitative analysis in the inset yielded a Total Energy Transfer (TET) value approaching 50%, confirming effective cross-linking and nanoscale embedding within the sponge matrix.

We further evaluated normalized FRET (N_FRET_) efficiency as a function of the acceptor-to-donor (A:D) fluorescence ratio (Figure 2c). In single-labeled controls (FITC-only or RBITC-only), N_FRET_ values remained low and scattered (Figure 2c­(i–ii)), consistent with the absence of FRET. In contrast, dual-labeled sponges showed clear N_FRET_ clustering at intermediate A:D ratios, peaking around 0.45, indicating efficient energy transfer and close spatial proximity between trypsin and BSA (Figure 2c­(iii)). Comparative N_FRET_ analysis across labeling conditions further confirmed these findings (Figure 2d­(i–iii)). Dual-labeled sponges consistently exhibited elevated N_FRET_ values, particularly in the 0.4–0.8 A:D range, while single-labeled controls showed minimal N_FRET_ signal.

These results provide strong evidence for the spatial colocalization and stable integration of trypsin within the BSA matrix. FRET microscopy confirms that the fabrication process enables enzyme embedding at the nanoscale, a prerequisite for preserving enzymatic activity and ensuring reproducibility in sponge-based digestion systems.

### Optimization and Characterization of the Structural, Mechanical, and Enzymatic Performance of BSA–Trypsin Sponges

To determine the optimal trypsin concentration for balancing structural robustness and catalytic efficiency, we fabricated sponges with increasing enzyme loadings (0.0015, 0.01, 0.4, and 1 mg/mL) and systematically characterized their structural, mechanical, and enzymatic performance.

We first assessed the water absorption capacity as an indirect measure of matrix porosity and hydrophilicity. All sponges were fabricated under identical foaming conditions (20 mM TW-20) to isolate the effects of trypsin content. Sponges with the lowest enzyme concentration of 0.0015 mg/mL exhibited the lowest water absorption of 3815 ± 848%, whereas those containing 0.4 and 1 mg/mL trypsin reached 3892 ± 507% and 4616 ± 577%, respectively (Figure 3a). These results suggest that higher enzyme content promotes denser cross-linking and more hydrophilic networks, enhancing water retention^48,49^ (Figure 3a).

**Figure 3:**
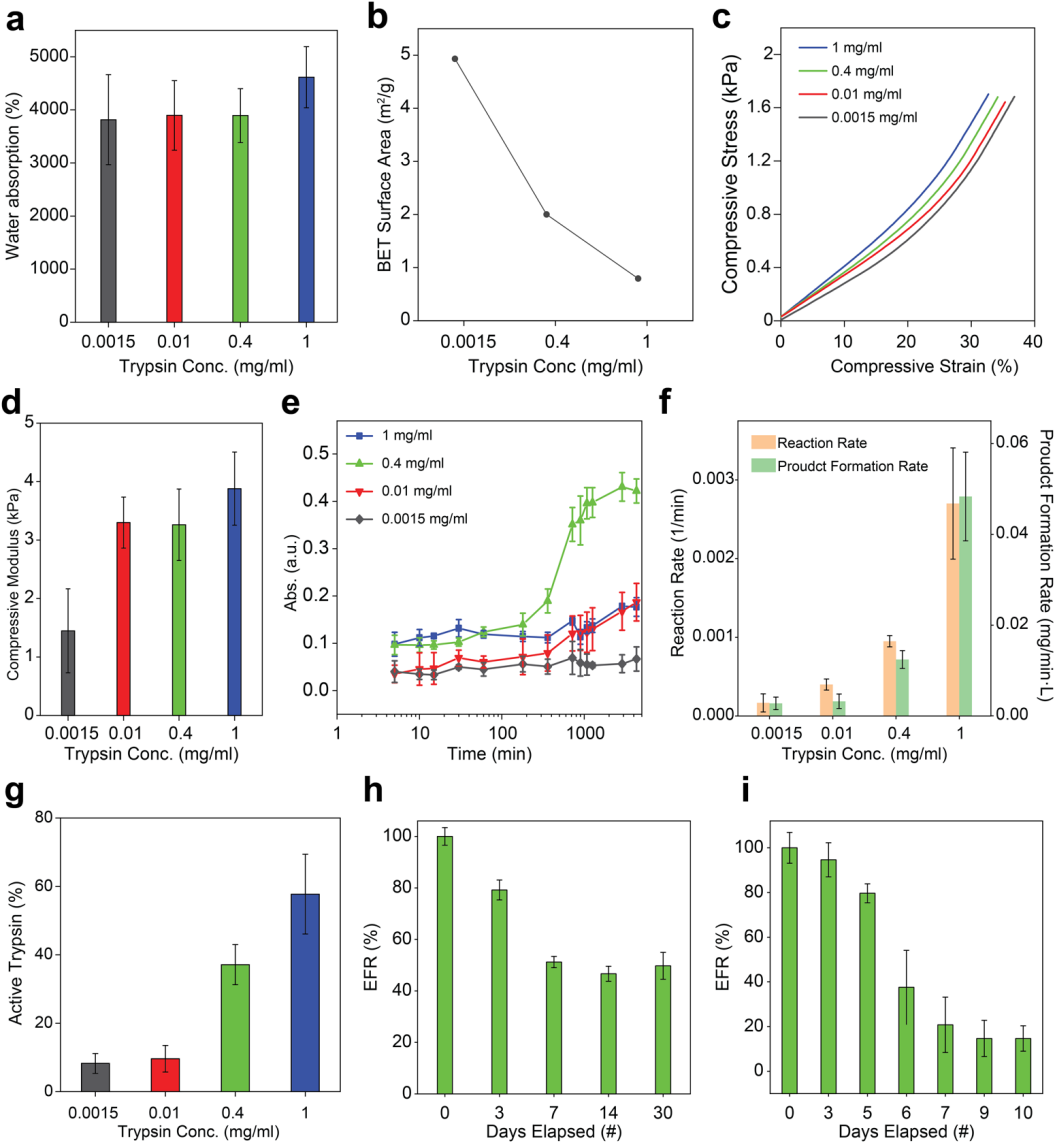
Optimization of BSA–trypsin sponges: Influence of trypsin concentration on structural, mechanical, and catalytic performance. (a) Water absorption capacity increases with trypsin concentration, indicating enhanced cross-linking and network density. (b) BET surface area decreases with increasing enzyme loading, consistent with reduced microporosity and denser matrix formation. (c) Stress–strain curves showing increased compressive resistance at higher trypsin concentrations. (d) Compressive modulus increases from 1.4 ± 0.7 kPa at 0.0015 mg/mL trypsin to 3.8 ± 0.6 kPa at 1 mg/mL, confirming stiffening due to increased cross-link density. (e) BAPNA cleavage, tracked by absorbance at λ_abs_ = 410 nm, shows rapid activity for 1 mg/mL trypsin, sustained kinetics for 0.4 mg/mL, and delayed activity at lower concentrations. (f) Initial reaction rate and product formation rate scale vs. trypsin content in the sponge; 1 mg/mL sponges exhibit the highest values, while 0.4 mg/mL supports prolonged catalysis. (g) The proportion of active trypsin retained within the sponge matrix increases with enzyme concentration, ranging from 8.2 ± 2.9% at 0.0015 mg/mL to 57.8 ± 11.7% at 1 mg/mL, confirming that higher initial trypsin concentrations facilitate greater enzymatic embedding and retention post-cross-linking. (h) Enzymatic function retention (EFR) over 30 days at 4 °C shows that 0.4 mg/mL sponges gradually declined and stabilized at ∼50% of their initial catalytic activity, indicating long-term storage stability. (i) Reusability over 10 digestion cycles shows an initial drop in EFR, followed by stabilization at approximately 60%, indicating sustained catalytic performance across multiple uses. All EFR values are expressed relative to the initial activity of freshly prepared free trypsin.

Surface area and porosity analyses supported these findings. BET measurements revealed a marked decrease in sponge surface area from 5.08 ± 0.31 m^2^/g at 0.0015 mg/mL trypsin, to 2.06 ± 0.09 m^2^/g at 0.4 mg/mL, and further to 0.81 ± 0.18 m^2^/g at 1 mg/mL (Figure 3b). HK and BJH models indicated corresponding reductions in micropore and mesopore volumes (Figures S2–S4), consistent with increased cross-linking density that reduces accessible pore space while enhancing matrix compactness and fluid retention.^50,51^

Compression tests further highlighted the effect of trypsin content on mechanical properties. Sponges with low enzyme concentrations were more deformable, with a modulus of 1.4 ± 0.7 kPa, while higher concentrations of 0.4 and 1 mg/mL yielded stiffer matrices with a modulus of 3.3 ± 0.6 and 3.8 ± 0.6 kPa, respectively, reflecting increased covalent cross-linking and enhanced structural integrity (Figures 3c, d).

To evaluate catalytic performance, Nα-Benzoyl-dl-arginine 4-nitroanilide hydrochloride (BAPNA) was used as a model substrate. Trypsin cleaves BAPNA to release p-nitroaniline, which was quantified spectrophotometrically at λ_abs_ = 410 nm over a three-day incubation at 37 °C. Sponges with 1 mg/mL trypsin showed a rapid increase in absorbance during the initial 300 min, indicating fast substrate cleavage. Sponges containing 0.4 mg/mL exhibited a slower but sustained reaction profile, while lower concentrations showed minimal catalytic activity (Figure 3e).

To better understand the enzymatic behavior of the sponges, initial reaction rates and product formation rates were quantified based on the linear region of absorbance over time. Sponges containing 1 mg/mL trypsin exhibited the highest activity, with an initial rate of 2.7 ± 0.7 × 10^–3^ min^–1^ and a product formation rate of 4.8 ± 0.9 × 10^–2^ mg/min·L. However, the reaction plateaued rapidly (Figure S5), likely due to the rapid consumption of the available BAPNA substrate, which limited further product formation.^52,53^ In comparison, sponges with 0.4 mg/mL trypsin showed more moderate but sustained behavior, with an activity rate of 9.5 ± 0.7 × 10^–4^ min^–1^, and a product formation rate of 1.2 ± 0.1× 10^–2^ mg/min·L. Notably, the increase in absorbance beyond ∼400 min for the 0.4 mg/mL formulation likely reflects progressive substrate diffusion into the less densely cross-linked matrix, allowing continued reaction over extended time scales. At the lowest enzyme concentration of 0.0015 mg/mL, the reaction rate dropped to 1.67 ± 1.15 × 10^–4^ min^–1^ and a product formation rate of 2.76 ± 1.38 × 10^–3^ mg/min·L, indicating insufficient enzymatic content for effective substrate cleavage (Figure 3f).

Enzyme retention within the sponge matrix was also quantified. The 1 mg/mL sponge retained 57.8 ± 11.7% of its active trypsin content postfabrication, compared to 37.1 ± 5.8% for 0.4 mg/mL and <10% for lower concentrations (Figure 3g). Retention was calculated by comparing the active enzyme concentration derived from product formation rates to the known specific activity of free trypsin. The higher retention observed at high enzyme loadings likely reflects reduced diffusion losses during fabrication, as well as the protective effect of denser cross-linked networks. These findings confirm that increased trypsin loading enhances enzymatic activity but may also promote faster substrate depletion and increased matrix rigidity, as evidenced by the higher initial reaction rates and greater total product formation (Figure 3f).

To assess long-term stability and reusability, the 0.4 mg/mL formulation was selected based on its favorable balance of structural integrity—evaluated by compressive modulus measurements and visual inspection—and sustained catalytic performance (Figure 3d-e and Figure S6). Sponges were stored at 4 °C for 30 days and tested at specific intervals—day 0, 3, 7, 14, and 30—by incubating them with BAPNA for 24 h, followed by absorbance measurement at λ_abs_ = 410 nm. Although a gradual decline in activity was observed, the enzymatic function retention (EFR) stabilized after 2 weeks, maintaining approximately 50 ± 5% of the initial activity by day 30 (Figure 3h). These findings demonstrate the structural stability and functional retention of the sponges under prolonged refrigeration and repeated use, underscoring their potential for long-term storage and on-demand use.

Reusability was further evaluated by subjecting sponges to ten consecutive 24-h digestion cycles with BAPNA. After each cycle, sponges were washed and reused. Although EFR declined during early cycles, ∼60 ± 3% of initial enzymatic function was retained by the tenth cycle (Figure 3i), highlighting the durability and applicability of the sponge system for repeated use in biocatalytic and proteomic workflows.

### Modulating Mechanical and Catalytic Performance via Acetic Acid-Mediated Cross-linking Control

To tune the balance between mechanical properties and enzymatic efficiency without altering enzyme concentration, we introduced AA during sponge synthesis to modulate Tyr-Tyr cross-linking. AA interferes with Ru­(II)-mediated dityrosine bond formation by generating carboxymethyl radicals, softening the matrix, and increasing accessibility of embedded trypsin sites.^27^

We first evaluated the water absorption capacity of sponges fabricated with 0.4 and 1 mg/mL trypsin under varying AA concentrations (0–1.5%). At 0.4 mg/mL trypsin, water uptake increased from 3892 ± 508% with no AA to a peak of 4632 ± 269% at 0.75% AA, then decreased at 1.5% AA to 4205 ± 874%. A similar trend was observed for 1 mg/mL trypsin, with maximum absorption of 4812 ± 299% at 0.75% AA, then decreased to 4587 ± 134% at 1.5% AA (Figure 4a). These results suggest that moderate AA concentrations improve sponge flexibility and swelling capacity by reducing cross-link density, while excessive AA leads to oversoftening and structural relaxation (Figure S6).

**Figure 4:**
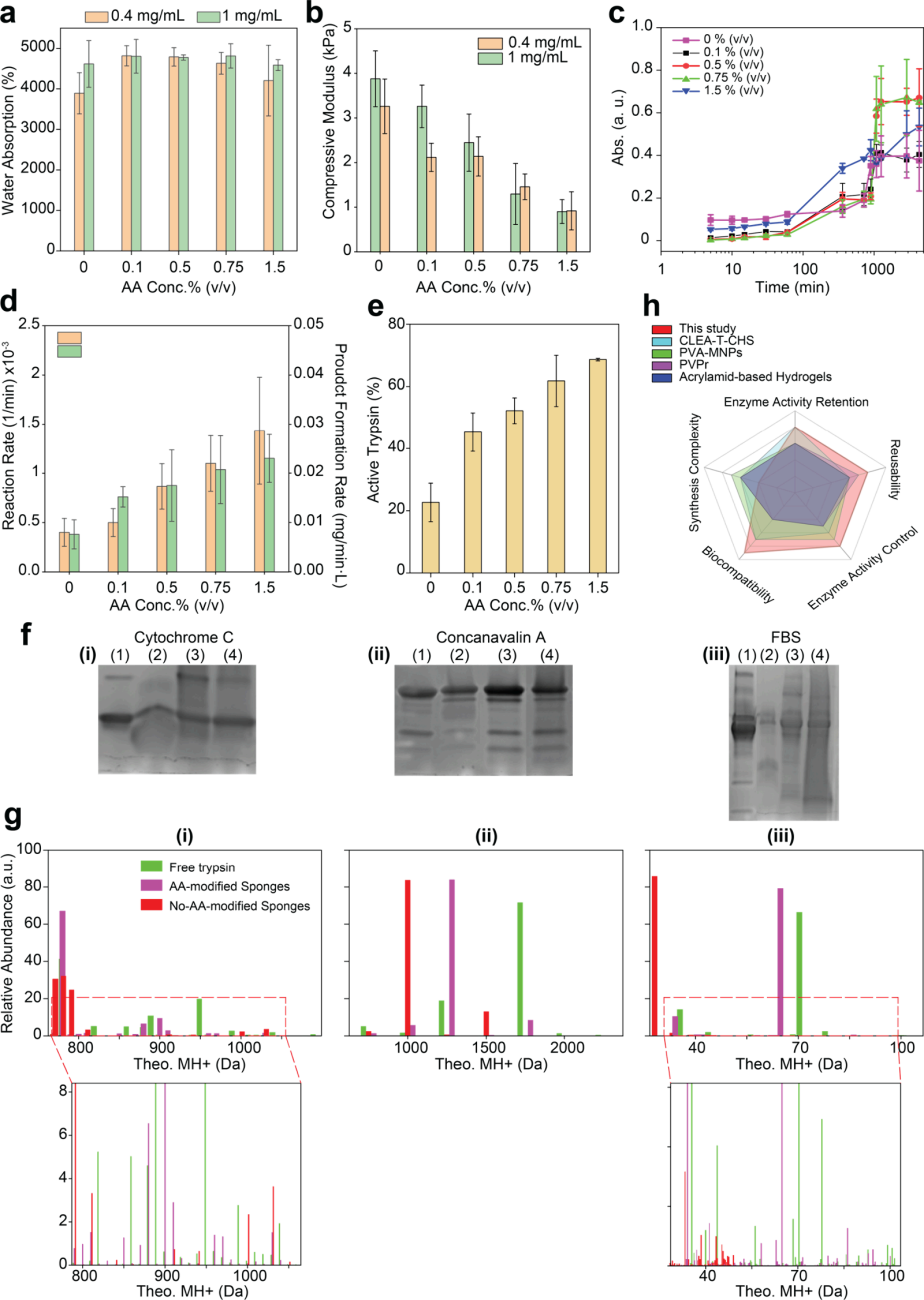
Effects of AA on the mechanical, swelling, and enzymatic properties of BSA–trypsin sponges. (a) Water absorption as a function of AA concentration (0–1.5%) for sponges with 0.4 and 1 mg/mL trypsin. Absorption increases with AA, peaking at 0.75%, and then decreases at 1.5%, indicating reduced structural integrity at high AA levels. (b) Compressive modulus decreases with increasing AA, confirming matrix softening due to reduced cross-linking. (c) BAPNA digestion profiles over 3 days for 0.4 mg/mL trypsin sponges. Absorbance at 410 nm increases by 0.5% and 0.75% AA, indicating enhanced catalytic activity; 1.5% AA shows reduced performance due to oversoftening. (d) Initial reaction rate and product formation rate increase with AA concentration, peaking at 1.5% AA, suggesting improved enzyme accessibility and substrate diffusion. (e) Active trypsin retention rises with AA, from 22.6% at 0% AA to 68.6% at 1.5% AA, consistent with increased enzyme exposure. (f) SDS-PAGE analysis of protein digestion by free trypsin, sponges without acetic acid (AA), and sponges containing 0.5% AA. Lane assignments: (1) Control (protein only, no trypsin), (2) free trypsin digestion, (3) non-AA-modified sponge digestion, and (4) AA-modified sponge digestion. (i) Cytochrome c shows extensive degradation by free trypsin, partial digestion by non-AA-modified sponges, and enhanced cleavage with AA-modified sponges. (ii) Concanavalin A digestion reveals faint bands with free trypsin, stronger bands with non-AA sponges, and reduced intensity with AA-modified sponges, indicating improved digestion. (iii) FBS digestion displays broad degradation by free trypsin, limited breakdown in non-AA sponges, and improved fragmentation with 0.5% AA. (g) LC-MS/MS peptide profiles of (i) Cytochrome c, (ii) Concanavalin A, and (iii) FBS. Non-AA sponges yield fewer low-mass peptides, whereas AA-modified sponges and free trypsin generate broader and more abundant peptide profiles, reflecting enhanced enzymatic digestion. (h) Radar chart comparing BSA–trypsin sponges with conventional hydrogel systems in terms of enzyme retention, activity control, reusability, and fabrication simplicity.

Mechanical testing supported these observations. Increasing AA reduced compressive stiffness in both 0.4 and 1 mg/mL trypsin formulations. In 0.4 mg/mL sponges, modulus values decreased from 3.2 ± 0.6 kPa at 0% AA to 2.1 ± 0.4 kPa at 0.5% AA, and to 0.9 ± 0.4 kPa at 1.5% AA. Similar trends were seen in 1 mg/mL sponges, with moduli dropping from 3.8 ± 0.6 to 2.4 ± 0.6 and 0.9 ± 0.2 kPa across the same AA range (Figure 4b). These results confirm AA’s role as a tunable modulator of network stiffness via controlled interference with cross-link formation.

To evaluate the influence of AA on enzymatic performance, we monitored the hydrolysis of BAPNA using sponges embedded with 0.4 mg/mL trypsin, previously identified as optimal for sustained activity. Over a three-day incubation at 37 °C, absorbance profiles revealed a progressive enhancement in enzymatic activity with increasing AA concentrations, peaking at 0.5% AA (Figure 4c). Product formation rates rose accordingly, from 7.6 ± 2.9 × 10^–3^ mg/min·L without AA to 1.75 ± 0.73 × 10^–2^ mg/min·L at 0.5% AA, and further to 2.30 ± 0.76 × 10^–2^ mg/min·L at 1.5% AA, where reaction rates began to plateau (Figure 4d). These enhancements correlated with the measured active trypsin content, which increased from 22.6 ± 6.1% at 0% AA to 52.1 ± 4.1%, 61.8 ± 8.2%, and 68.6 ± 0.4% for sponges with 0.5%, 0.75%, and 1.5% AA, respectively (Figure 4e). Together, these findings indicate that acetic acid effectively modulates cross-linking density to increase enzyme accessibility and catalytic output.

To assess the practical digestion capabilities of the AA-modified sponges, we evaluated their proteolytic efficiency across three representative substrates of increasing structural complexity: Cytochrome c (12 kDa), Concanavalin A (26 kDa), and FBS, a complex mixture of proteins. The sponges were prepared with 1 mg/mL trypsin, as this concentration provides increased availability of active trypsin sites, reaching approximately 60% activity compared to 40% at 0.4 mg/mL (Figure 3g). This results in more efficient protein cleavage and a higher hydrolysis rate, essential for digesting complex protein substrates. Among the AA-modified formulations, 0.5% AA was selected based on its optimal balance between mechanical softness, water absorption capacity, and enzymatic efficiency (Figures 4a-e). After enzymatic digestion, SDS-PAGE and LC-MS/MS were employed to qualitatively assess protein degradation, providing a visual comparison of digestion profiles across conditions—free trypsin, non-AA sponges, and 0.5% AA-modified sponges.

Across all three substrates, free trypsin and AA-modified sponges consistently demonstrated enhanced proteolytic activity compared to their non-AA counterparts. In SDS-PAGE, intact protein bands in the control lanes remained sharp and prominent, indicating limited digestion, whereas both free trypsin and AA-modified sponges resulted in substantial band degradation and the appearance of lower molecular weight fragments. For Cytochrome c and Concanavalin A, the AA-modified sponges closely mirrored the digestion pattern observed with free trypsin, exhibiting significantly reduced intact bands and a broader smear of fragments, indicative of extensive cleavage. In contrast, non-AA sponges showed only partial degradation, with many high-molecular-weight bands remaining intact (Figure 4f­(i-ii)).

These observations were further supported by LC–MS/MS analysis, which provided a quantitative profile of peptide generation. Peptide mass distributions from AA-modified sponges closely aligned with those of free trypsin, producing a broad range of fragments, particularly in the low- and midmass regions (600–1400 Da). In contrast, non-AA sponges generated fewer peptides, with a noticeable underrepresentation of low-mass fragments (<800 Da), suggesting limited cleavage due to steric hindrance within the densely cross-linked network (Figure 4g­(i-ii)). This trend extended to the digestion of FBS, where free trypsin and AA-modified sponges effectively reduced high-molecular-weight protein bands and generated a diffuse pattern of low-mass fragments on SDS-PAGE (Figure 4f­(iii)). The corresponding mass spectrometry data confirmed this profile, with broad and intense peptide distributions reflecting efficient digestion. Conversely, sponges without AA retained many intact bands and yielded a narrower, less intense peptide mass spectrum (Figure 4g­(iii)). To quantitatively assess the similarity of cleavage profiles, Pearson correlation coefficients were calculated between the relative peptide abundances. For sponges prepared with 0.5% AA, high correlations were observed across all substrates (Concanavalin A: *r* = 0.954; Cytochrome c: *r* = 0.883; FBS: *r* = 0.963), demonstrating that AA-modulated sponges closely replicate the cleavage specificity of free trypsin. In contrast, sponges fabricated without AA exhibited substantially lower correlations (Concanavalin A: *r* = −0.474; Cytochrome c: *r* = 0.500; FBS: *r* = −0.011), underscoring the importance of cross-linking modulation for consistent proteolytic performance. Collectively, these findings demonstrate that AA-mediated modulation of cross-linking enhances enzymatic accessibility without compromising matrix stability.

## Discussion

This study presents a robust strategy for engineering BSA–trypsin-based sponges that overcome key limitations of free enzymes and conventional immobilization platforms. Through covalent cross-linking of trypsin into a BSA matrix, we achieved enhanced enzymatic stability, prolonged activity, and mechanical resilience, establishing a scalable platform for proteolytic applications.

The developed BSA–trypsin sponge platform is particularly suited for applications that demand extended enzyme reuse, consistent proteolytic performance across multiple digestion cycles, and prolonged storage stability without refrigeration. Such contexts include industrial protein hydrolysate production,^54^ therapeutic protein processing pipelines,^55^ and high-throughput proteomic workflows where enzyme immobilization has demonstrated clear economic and operational advantages.^56^ While this system is not intended to replace freshly prepared free trypsin in routine laboratory workflows—where inexpensive, single-use enzymes are adequate—it provides a robust and reusable alternative for settings where long-term functionality and reduced enzyme consumption are critical.

BSA, owing to its high molecular weight (66 kDa) and the presence of accessible tyrosine residues on its surface, served as both a structural scaffold and cross-linking partner for trypsin (23.8 kDa), which lacks the intrinsic stability for reuse on its own.^25,57,58^ The light-induced Tyr-Tyr cross-linking between BSA and trypsin yielded a mechanically robust, porous matrix that retained significant enzymatic activity over time and multiple uses.

FRET microscopy confirmed the nanoscale integration of trypsin within the BSA network, as indicated by overlapping FITC–BSA and RBITC–trypsin signals and elevated N_FRET_ values (Figure 2). This close spatial proximity ensured optimal enzyme accessibility and structural coherence, which are essential for long-term catalytic performance.

Systematic modulation of trypsin concentration revealed that higher enzyme loadings (up to 1 mg/mL) enhanced matrix density and water absorption capacity (Figure 3a) but led to faster substrate depletion and a shorter catalytic window. In contrast, sponges with 0.4 mg/mL trypsin exhibited sustained enzymatic activity over extended periods, achieving a favorable balance between catalytic performance and mechanical integrity (Figure 3e). The optimized structure retained ∼60% activity after 10 reuse cycles and ∼50% after 30 days of refrigeration (Figure 3h–i), substantially outperforming free trypsin, which lost over 50% activity within only 4 h at 37 °C and retained only ∼21% after 4 weeks at 4 °C.^59,60^

To enhance digestion efficiency without increasing enzyme concentration, we introduced AA as a cross-linking modulator.^61^ AA interfered with dityrosine bond formation, softening the matrix, improving water uptake, and exposing more active enzyme sites. This was evidenced by increased swelling capacity, reduced compressive modulus, and a 3-fold increase in product formation rate at 0.5% AA compared to untreated sponges (Figures 4a–d). Enzyme retention also improved, reaching ∼69% active trypsin at 1.5% AA (Figure 4e).

Building on these improvements in matrix properties, AA-modified sponges exhibited markedly enhanced digestion of diverse protein substrates, including Cytochrome c, Concanavalin A, and FBS. SDS-PAGE and LC–MS/MS analyses revealed peptide fragmentation profiles from AA-treated sponges that closely mirrored those of free trypsin, in contrast to the limited digestion observed in non-AA controls (Figures 4g–h). For valid comparison, the free trypsin concentration used in SDS-PAGE and LC–MS/MS assays was matched to the amount embedded in the sponges, ensuring that digestion differences reflected matrix performance, not enzyme dosage. These results confirm the role of AA in modulating cross-link density to enhance enzymatic access and digestion performance across simple and complex proteins.

To further evaluate the intrinsic effectiveness of the embedded enzyme, no reduction or alkylation steps were employed in the digestion protocols. The highly porous architecture and large surface area of the BSA–trypsin-based sponges would facilitate efficient substrate diffusion and enzymatic access even in the absence of reducing agents. This approach underscores that the observed digestion performance reflects the combined effects of matrix permeability and enzyme accessibility, rather than enhanced substrate unfolding.

Additionally, parameters such as pore size distribution, density, and hydrophilicity provide useful indicators of potential enzyme accessibility and retention. However, the overall morphology and connectivity of the sponge network likely have a more significant influence on substrate diffusion and interaction with active sites. Each of the proteins investigated in this study—Cytochrome c, Concanavalin A, and the complex mixture of FBS—possesses distinct molecular sizes, tertiary structures, and surface hydrophilicity, which can impact their ability to penetrate the porous matrix and engage with the cross-linked trypsin. For example, larger or more hydrophilic proteins may experience steric hindrance or slower diffusion within densely cross-linked regions, whereas smaller proteins can more readily access internal enzymatic sites. These factors, in combination with matrix swelling and cross-linking density, are likely to underlie the substrate-specific digestion efficiencies observed across our assays.

Compared to traditional immobilization systems, our sponges demonstrated superior long-term reusability and storage stability. To contextualize these advantages, representative enzyme-support systems were evaluated based on several key performance criteria, including activity retention, multicycle reusability, tunability of enzyme activity through matrix properties, biocompatibility, and synthesis complexity (Figure 4h). Although all platforms are broadly categorized as immobilized enzyme systems, they vary significantly in matrix composition, enzyme loading capacity, substrate scope, reaction conditions, and characterization methods. For example, trypsin cross-linked aggregates with chitosan (CLEA–T–CHS) retained only 64% of their activity after five reuse cycles, with performance loss attributed to handling-related stress.^60^ Similarly, trypsin immobilized on glutaraldehyde-activated poly­(vinyl alcohol)-coated magnetic nanoparticles (PVA–MNPs) retained just 50% of its activity after 12 days at 4 °C and 56% after eight digestion cycles.^62^ Other platforms, such as PVPr-based or acrylamide-based hydrogels, showed improved thermal stability but suffered from lower enzyme accessibility or diffusion constraints due to dense or hydrophobic cross-linking networks.^63^

Beyond these conventional supports, polyurethane-based sponges have also been explored for enzyme immobilization. For example, glycosylated fungal enzymes such as aminoacylase and phytase were covalently bound within polyurethane foams, achieving high enzyme loadings of up to 200 mg protein per gram of foam and retained activities of 60–100%. However, this approach relied on isocyanate prepolymers, which pose toxicity concerns and require careful temperature control during exothermic polymerization, limiting compatibility with sensitive enzymes and biomedical applications.^64^ Separately, immobilized parathion hydrolase sponges were developed for detoxifying organophosphate pesticides. These systems demonstrated good liquid absorption and multicycle reusability for pesticide degradation but were designed primarily for small-molecule hydrolysis and not for proteolytic digestion of protein substrates.^65^

In contrast, our system retained ∼50% activity after 30 days and ∼60% after 10 reuse cycles without enzyme leaching (Figure S7). Its fabrication relies on biocompatible, naturally derived components and a photoactivated, additive-free cross-linking chemistry performed in a single step. This approach avoids toxic reagents, simplifies processing, and improves scalability, making it suitable for biomedical, proteomic, and industrial applications.

Importantly, while many conventional systems are technically biocompatible, their use is limited by complex synthesis procedures, chemically harsh conditions, or constrained enzyme loading. Our AA-modulated sponges overcome these limitations by allowing precise tuning of matrix stiffness and enzyme accessibility without altering the enzyme content. The resulting platform enables efficient digestion, simplified fabrication, and robust mechanical properties in a reusable and scalable format.

Future studies may explore incorporating multiple enzymes to enable cascade reactions, expanding the utility of these sponges in high-throughput proteomics and synthetic biology workflows. Additionally, tuning the matrix with alternative modulators beyond AA could allow more precise control over network architecture for specialized applications, such as bioremediation or therapeutic delivery. While this study focused on 4 °C storage as a standard preservation condition, evaluating room temperature stability could provide valuable additional insights. Additionally, this study did not directly compare sponge performance to trypsin reconstituted in 1% acetic acid, which is commonly used in proteomics. Future work will include this benchmark to better contextualize the advantages of the sponge system. Moreover, while this study focused on uniformly sized sponges produced in fixed molds, a systematic investigation of how sponge size and geometry influence enzymatic efficiency, diffusion dynamics, and mechanical properties would be valuable for future optimization, particularly in application-specific or scaled-up formats. These directions will further strengthen the platform’s utility across a broad range of biocatalytic systems.

## Conclusion

In this study, we developed a structurally tunable, enzymatically active BSA–trypsin sponge platform that addresses key limitations of free enzymes and traditional immobilization methods. By leveraging light-induced covalent cross-linking and acetic acid–mediated modulation of network density, we achieved precise control over sponge porosity, mechanical integrity, and enzyme accessibility. This platform maintained proteolytic performance comparable to freshly prepared free trypsin while offering enhanced operational stability, prolonged activity retention, and reusability over multiple digestion cycles.

Importantly, the system enabled efficient digestion of diverse protein substrates, including complex mixtures such as FBS, with cleavage profiles comparable to those of free trypsin. These features were achieved without requiring high enzyme loadings or harsh immobilization chemistries, positioning the platform as a scalable, biocompatible, and reusable solution for applications in proteomics, biocatalysis, and beyond.

Beyond proteomics workflows, enzyme reusability and prolonged operational stability are critical in industrial applications such as large-scale production of protein hydrolysates for food applications, therapeutic protein processing, and continuous-flow digestion systems used in bioreactors and pharmaceutical manufacturing.^66,67^ In these contexts, BSA–trypsin-based sponges that retain activity over extended timeframes can reduce costs, minimize downtime, and improve process consistency. Future extensions of this platform may include multienzyme integration, programmable degradation—achieved, for example, by introducing cleavable cross-linkers that respond to pH, light, or specific enzymes, or adaptation for therapeutic or diagnostic use. Collectively, this work introduces a versatile approach to enzyme stabilization and matrix design with broad relevance across biological and materials science domains.

## Methods Section

### BSA-Trypsin-Based Sponge Synthesis

Trypsin at final concentrations of 0.0015, 0.01, 0.4, and 1 mg/mL was mixed with BSA at a final concentration of 132 mg/mL in 20 mM TW-20, prepared in TRIS (20 mM Tris, 150 mM NaCl, pH ∼ 7.4). From this mixture, 450 μL was combined with 30 μL of Ru­(II)­(bpy)_3_^+2^ (6.67 mM) and 30 μL of APS (1 M) in a 15:1:1 volume ratio. The resulting mixture was homogenized using a Bio-Gen PRO200 homogenizer at 15K rpm for 2.5 min at room temperature to produce the foam. The foam was then transferred into an 8 mm diameter cylindrical mold and exposed to white LED light at 1000 lx for 30 min, initiating covalent cross-linking between the tyrosine residues of BSA and trypsin, thereby forming the sponge structure. Finally, the sponges were washed three times with TRIS at room temperature to remove residual TW-20 and other intermediates. To control the cross-linking density within BSA-trypsin-based sponges, phosphate buffer solutions were prepared containing ∼10 mM NaH_2_PO_4_, ∼150 mM NaCl, and 20 mM TW-20, with varying concentrations of AA (0.1, 0.5, 0.75, and 1.5% v/v). The pH of these solutions was adjusted to ∼7.4 using 1 M KOH. BSA and trypsin were then dissolved in the different buffers to achieve final concentrations of 132 mg/mL BSA and either 0.4 or 1 mg/mL trypsin. The protein mixture, APS, and Ru­(II)­(bpy)_3_^+2^ were combined in a volume ratio of 15:1:1, homogenized, and the resulting foam was exposed to white light to initiate the covalent cross-linking process in the presence of AA. The formed sponges were washed with TRIS at room temperature to remove any remaining TW-20, AA, and other intermediates.

### ATR-FTIR

The ATR-FTIR spectra were obtained for both trypsin solution and trypsin-based sponges containing 1 mg/mL of trypsin, using a Nicolet iS50 FTIR instrument in ATR mode with a round diamond (Type IIa crystal). Sixteen scans were recorded for each sample with a resolution of 8 cm^–1^. The different conformations of the main secondary structures of trypsin in solutions and sponges, including intramolecular β-sheets (1610–1630 cm^–1^), random coil (1640–1648 cm^–1^), α-helix (1648–1660 cm^–1^), and β-turns (1660–1689 cm^–1^), were analyzed by spectral deconvolution of the Amide I band (1600–1700 cm^–1^) using OMNIC FTIR software.

### Water Absorption Measurement

BSA-trypsin-based sponges prepared with different concentrations of trypsin (0, 0.0015, 0.01, 0.4, and 1 mg/mL) were immersed in TRIS for 24 h at 4 °C to ensure complete swelling. After immersion, the sponges were removed from the TRIS solution, gently blotted with filter paper to remove excess buffer, and weighed to obtain their wet weight (W_wet_). The sponges were then washed three times with ddH_2_O at RT, freeze-dried, and weighed again to obtain their dry weight (W_dry_). The water absorption ratio was calculated using the following equation.

Wwet−WdryWdry∗100

### Quantitative Porosimetry of BSA-Trypsin-Based Sponges

The micropore and mesopore size distributions of the BSA-trypsin-based sponges were analyzed using nitrogen adsorption and desorption at 77 K with a 3Flex instrument (Micromeritics, Norcross). Microporosity was determined from the adsorption curves using the HK model, assuming a cylindrical pore geometry. The mesopore size distribution was calculated from the adsorption curves using the BJH model. In both HK and BJH analyses, pore size distributions were calculated computationally by the instrument software based on the adsorption–desorption isotherms and represent the contributions of different pore diameters within the same sample, rather than experimentally varied pore widths. The *S*_BET_ was determined using the 5-point BET method. For each nitrogen sorption analysis, 30 sponges were pooled to obtain sufficient material for measurement.

### Mechanical Characterization

For compressive testing, BSA-trypsin-based sponges prepared with varying concentrations of trypsin (0.0015, 0.01, 0.4, and 1 mg/mL) were prepared into cylindrical shapes, washed with TRIS, and soaked in TRIS for 3 h to equilibrate. Another set of sponges was prepared with 0.4 or 1 mg/mL trypsin and varying concentrations of AA (0.1%, 0.5%, 0.75%, and 1.5% v/v). The dimensions of the sponges were measured using a digital Vernier caliper (NEIKO). Compression tests were conducted with a Dynamic Mechanical Analyzer (DMA, Anton Paar) using a ramp linear force profile, where 1000 data points were collected over a constant duration, with an interval of 0.6 s up to 60 s. The applied force started at 0.01 N and increased linearly to 0.1 N. The compressive modulus was calculated from the linear region of the resulting stress–strain curves.

### FRET Microscopy for BSA-Trypsin-Based Sponges’ Visualizations

To visualize the spatial distribution of BSA and trypsin within the sponge matrix, both proteins were fluorescently labeled prior to sponge formation. BSA was dissolved in 0.1 M carbonate–bicarbonate buffer (pH ∼ 9–9.5) to a final concentration of 2 mM and labeled with 1 mM FITC, while trypsin was dissolved in the same buffer to 4 mg/mL and labeled with 2 mg/mL RBITC, acheving fluorophore-to-protein (F/P) ratio of 0.5. This approach aims to achieve a F/P ratio between 0.3 and 1.0, balancing fluorescence intensity and protein functionality, following established labeling protocols.^68^ Both solutions were incubated in the dark at room temperature for 1.5 h, dialyzed against deionized water for 48 h using 6–8 kDa MWCO dialysis bags to remove excess dye, and then lyophilized at 77 K and 0.016 mbar. The labeled trypsin-RBITC was mixed with BSA-FITC in 20 mM TW-20 to obtain final concentrations of 0.4 mg/mL trypsin and 132 mg/mL BSA. This mixture (450 μL) was combined with 30 μL of Ru­(bpy)_3_^2+^ (6.67 mM) and 30 μL of APS (1 M) in a 15:1:1 volume ratio, foamed as described previously, and transferred to a 12-well plate. The foam was exposed to white LED light for 30 min using a 400–450 nm violet filter to facilitate Ru­(II)-mediated photo-cross-linking between tyrosine residues, while minimizing photobleaching. After curing in the 12-well plate, circular sponges (8 mm diameter, 2 mm thickness) were cut directly from the wells using a cylindrical punch cutter and then washed three times with TRIS (pH ∼ 7.4) to remove residual surfactants and unreacted compounds. Confocal FRET microscopy was performed using a Zeiss LSM 710 AxioObserver equipped with a Plan-Apochromat 20×/0.8 M27 objective to evaluate protein localization. FITC was excited with a 488 nm laser (0.1% power) and detected in the 484–540 nm range, while RBITC was excited at 543 nm (2.0% power) and detected in the 565–797 nm range, using PMT detectors at 750 gain. Main beam splitter 458/543 and pinhole settings of 2.93 AU (FITC) and 3.27 AU (RBITC) were used. Imaging was conducted in z-stack mode (32 slices over 124 μm, 4 μm steps), with a resolution of 1024 × 1024 pixels (0.42 μm/pixel) and a pixel dwell time of 1.27 μs. FRET signals were analyzed using ZEN software, with thresholding and colocalization analysis confirming that energy transfer between FITC (donor) and RBITC (acceptor) occurred due to proximity, validating the structural integration of BSA and trypsin within the sponge matrix.

### Quantification of Enzymatic Activity in BSA-Trypsin-Based Sponges Using Absorbance Kinetics and Product Formation Analysis

BSA-trypsin-based sponges were prepared with different concentrations of trypsin (0.0015, 0.01, 0.4, and 1 mg/mL). Another set of sponges was prepared with 0.4 mg/mL trypsin and varying concentrations of AA (0.1%, 0.5%, 0.75%, and 1.5% v/v).

These sponges were placed in a custom-designed scaffold system (Figure S8) that securely held them in place within a 12-well plate containing 2 mM BAPNA solution (∼pH 8). The scaffold consisted of cylindrical molds with open bottoms and perforated tops and sides, allowing full diffusion of the solution around and through the sponges to ensure uniform exposure during the enzymatic assay. The system was incubated at 37 °C with a mixing speed of 40 rpm. Over a period of 3 days, the absorbance of the solution was monitored at 410 nm using a Tecan Infinite 200 PRO spectrophotometer to evaluate the enzymatic digestion of BAPNA, indicated by the release of the yellow chromogenic product, p-nitroaniline. All measurements were performed in triplicate

To calculate the reaction rate, the linear slope of the graph plotting absorbance as a function of time in minutes was determined. This slope reflects the change in absorbance per unit time, providing a quantitative measure of enzymatic activity in units of (min^–1^).

Then, the concentration of the product was calculated by using the Beer–Lambert’s Law:

$$Product\;concentration\;\left(mM\right)=\frac{A*1000}{\varepsilon *l}$$

Where:

*A* - is the absorbance measured at 410 nm.

1000 is the conversion factor from moles per liter to millimoles per liter.

*ε* - is the molar extinction coefficient of p-nitroaniline at 410 nm, which is 8800 L/mol·cm.

*l* - is the optical path length of the well, set at 0.9 cm.

Subsequently, the linear slope of the graph plotting product concentration versus time (in mM/min) was determined, representing the rate of p-nitroaniline formation. To express this in mass units (mg/min·L), the slope was multiplied by the molecular weight of p-nitroaniline (138.12 g/mol) and adjusted for unit conversions:

$$Product\;formation\;rate\;\left(\frac{mg}{\min\cdot L}\right)=slope\;\left(\frac{mM}{\min}\right)*\mathrm{molecular}\;\mathrm{weight}\;\left(\frac{g}{mol}\right)$$

### Calculation of Enzyme Specific Activity and Active Enzyme Percentage

To calculate the percentage of active trypsin in the sponge, the specific activity of free trypsin was calculated by mixing different concentrations of BAPNA (0.005, 0.01, 0.015, 0.02, and 0.04 mM) and 0.4 mg/mL of trypsin, while keeping the entire setup on ice. The enzyme–substrate reaction was allowed to proceed for 25 min at 37 °C, a duration optimized in preliminary trials to remain within the linear kinetic range while avoiding substrate saturation. The absorbance of the reaction mixture was measured, and the product concentration was calculated using Beer–Lambert’s Law. From this, the product formation rate of free trypsin was determined and subsequently used to calculate its specific activity, which served as a reference for evaluating enzyme performance within the sponge matrix. The specific activity was calculated using the following equation:

$$Specific\;activity\;of\;tryp\sin\;\left(\frac{\mu mol}{\min}/mg\right)=\frac{Product\;formation\;rate\;of\;free\;tryp\sin\;\left(\frac{\mu mol}{\min*mL}\right)}{Concentration\;of\;free\;tryp\sin\left(mg/mL\right)}$$

This calculation provided the enzyme’s catalytic efficiency in solution and was used to estimate the concentration of active trypsin retained within the sponge using the following equation:

$$Active\;tryp\sin\;concentration\;\left(mg/mL\right)=\frac{Product\;formation\;rate\;of\;the\;sponge\;\left(\frac{\mu mol}{\min*mL}\right)}{Specific\;activity\;of\;tryp\sin\left(\frac{\mu mol}{\min}/mg\right)}$$

To express the amount of active trypsin retained within the sponge relative to the initially added trypsin, the active enzyme percentage was calculated using the following equation:^69^

$$Active\;tryp\sin\;percentage\;\left(\%\right)=\frac{Active\;tryp\sin\;concentration\;\left(mg/mL\right)}{Initial\;tryp\sin\;concentration\;\left(mg/mL\right)}*100$$

### Enzymatic Reusability of BSA-Trypsin-Based Sponges

To evaluate the longevity and repeated-use effectiveness of our enzymatic sponges, we conducted multiple cycles of BAPNA (2 mM, pH ∼ 8) digestion. BSA-trypsin-based sponges prepared with 0.4 mg/mL of trypsin were initially soaked in a BAPNA solution for 24 h, allowing for enzymatic interaction. Following this incubation, the sponges were thoroughly washed in TRIS for 4 h to remove any residual BAPNA and byproducts. Subsequently, the sponges were resoaked in fresh BAPNA solution (2 mM, pH ∼ 8), initiating a new digestion cycle. This process was repeated over a span of 10 days.

The retention of enzymatic function was quantitatively assessed after each 24-h digestion period. To measure enzymatic function retention, the absorbance of the solution was recorded, and the following formula was used to calculate the percentage of retained enzymatic activity:^70^

$$Retention\;of\;enzymatic\;function\;\left(\%\right)=\frac{A_{time\;t}}{A_{initial}}*100$$

Where:

*A*_*time t*_ - is the absorbance measured after each 24-h cycle,

*A*_*initial*_ - is the maximum absorbance observed during the first cycle, serving as a baseline for maximal enzymatic activity.

### Assessment of BSA-Trypsin-Based Sponge Longevity Under Refrigerated Storage

To evaluate the stability and longevity of our enzymatic sponges under refrigerated conditions, BSA-trypsin-based sponges prepared with 0.4 mg/mL were prepared and stored at 4 °C. Sets of sponges were used for enzymatic activity assays after their preparation and other sets were used after storage intervals of three, seven, 14, and 30 days. Each set was soaked in a BAPNA solution (2 mM, pH = ∼8) for 24 h for enzymatic digestion.

As before, the retention of enzymatic function was quantitatively assessed after each storage interval to determine the sponges’ effectiveness over time.

$$Retention\;of\;enzymatic\;function\;\left(\%\right)=\frac{A_{time\;t}}{A_{initial}}*100$$

Where:

*A*_*time t*_ - is the absorbance measured after each storage interval.

*A*_*initial*_ - is the maximum absorbance observed for the first set, serving as a baseline for maximal enzymatic activity.

### Evaluating Protein Digestion Efficiency of BSA-Trypsin-Based Sponges Using SDS-PAGE

BSA–trypsin-based sponges were prepared with 1 mg/mL trypsin, both without AA and with 0.5% AA, alongside free trypsin controls. The sponges were soaked in solutions of Cytochrome c and Concanavalin A at a concentration of 0.5 mg/mL (∼pH = 8), as well as with FBS diluted 1:100 from the commercial stock solution (Sigma). All samples were incubated for 12 h at 37 °C with agitation at 40 rpm, without any predenaturation of the proteins.

Following incubation, the protein digestion efficiency was analyzed using SDS-PAGE on 4–15% precast polyacrylamide gels. Electrophoresis was conducted at 150 V for initial migration and then reduced to 100 V for separation. Gels were stained with Biosafe Coomassie and visualized using a ChemiDoc MP imaging system.

### Analyzing Proteolytic Performance of BSA-Trypsin-Based Sponges Using LC-MS/MS

Protein samples previously digested using BSA–trypsin-based sponges (with and without 0.5% AA) or free trypsin were further processed for LC-MS/MS analysis to identify and quantify the resulting peptide fragments. 100 μL of protein samples were brought to 8.5 M Urea, 100 mM ammonium bicarbonate, and 10 mM DTT. The samples were reduced at 60 °C for 30 min, modified with 35.2 mM iodoacetamide in 100 mM ammonium bicarbonate at room temperature for 30 min in the dark, and subjected to a 10 kDa molecular weight cutoff. The resulting filtrate was desalted using an Oasis HLB 96-well μElution Plate (Waters), dried, and resuspended in 0.1% trifluoroacetic acid. Detergent was removed using SCX stage tips (homemade from Empore cation disks), and the samples were dried and resuspended in 0.1% formic acid in 2% acetonitrile. The resulting peptides were analyzed by LC-MS/MS using a Q Exactive Plus mass spectrometer (Thermo) equipped with a capillary HPLC system (Ultimate 3000, Thermo Scientific). Peptides were loaded in solvent A (0.1% formic acid in water) onto a homemade capillary column (30 cm, 75 μm ID) packed with Reprosil C18-Aqua (Dr. Maisch GmbH, Germany) and separated using a linear gradient of 5% to 28% solvent B (99.99% acetonitrile with 0.1% formic acid) over 120 min, followed by a 15 min gradient from 28% to 95%, and 15 min at 95% solvent B, at a flow rate of 0.15 μL/min. Mass spectrometry was performed in positive mode (*m*/*z* 300–1500) with a resolution of 70,000 for MS1 and 17,500 for MS2, using a top-10 data-dependent acquisition method. Fragmentation was performed via higher-energy collision dissociation with a normalized collision energy of 25. AGC target values were 3 × 10^6^ for MS1 and 1 × 10^5^ for MS2, with an intensity threshold of 1 × 10^4^ and a dynamic exclusion duration of 20 s. The raw MS data were processed using Proteome Discoverer 2.4 (Thermo), with Sequest used to search against the protein database, allowing a mass tolerance of 20 ppm for precursors and 0.05 Da for fragments. Oxidation of methionine and N-terminal acetylation were set as variable modifications, while carbamidomethylation on cysteine was set as a static modification. The minimum peptide length was six amino acids, and up to two missed cleavages were allowed. Peptide-level false discovery rates were filtered to 1% using the target-decoy approach, and quantification was performed using label-free quantification within the same software.

## Supplementary Material



## Notes

ATR–FTIR spectra, nitrogen sorption data, pore size analyses, BAPNA digestion kinetics, sponge morphology images, protein leaching assays, and scaffold design for enzymatic tests (PDF)
