# Supplementary material for: Bovine Serum Albumin–Trypsin Sponges for Enhanced Enzymatic Stability and Protein Digestion Efficiency
Source: ACS Appl Bio Mater. 2025 Sep 19;8(10):8889–902. doi: 10.1021/acsabm.5c01038 (PMC12541704; doi:10.1021/acsabm.5c01038)
Supplement: Supplementary file 1 [file mt5c01038_si_001.pdf]

SUPPORTING INFORMATION

Bovine Serum Albumin–Trypsin Sponges for Enhanced Enzymatic Stability and Protein Digestion Efficiency

*Maria Kaeek[1], Luai R. Khoury[1]**

[1]Technion Israel Institute of Technology, Department of Materials Science and Engineering, Haifa, 32000, Israel.

* Corresponding author: Luai R. Khoury, luaikh@technion.ac.il

Supplementary Figures

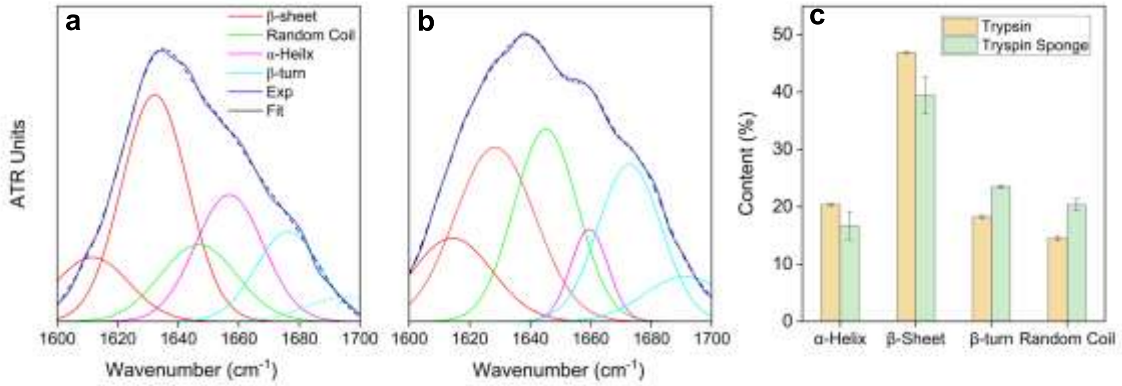

Figure S1. ATR–FTIR deconvolution of secondary structures in free and sponge-embedded trypsin. (a) Deconvoluted ATR–FTIR spectrum (Amide I region) of free trypsin in TRIS, showing α-helix, β-sheet, β-turn, and random coil components. **(b)** Corresponding deconvoluted spectrum of trypsin incorporated within the sponge matrix, revealing similar secondary structure distributions. **(c)** Quantitative comparison of the secondary structure composition between trypsin in solution and trypsin incorporated within the pure trypsin-based sponge, confirming structural preservation post-fabrication.

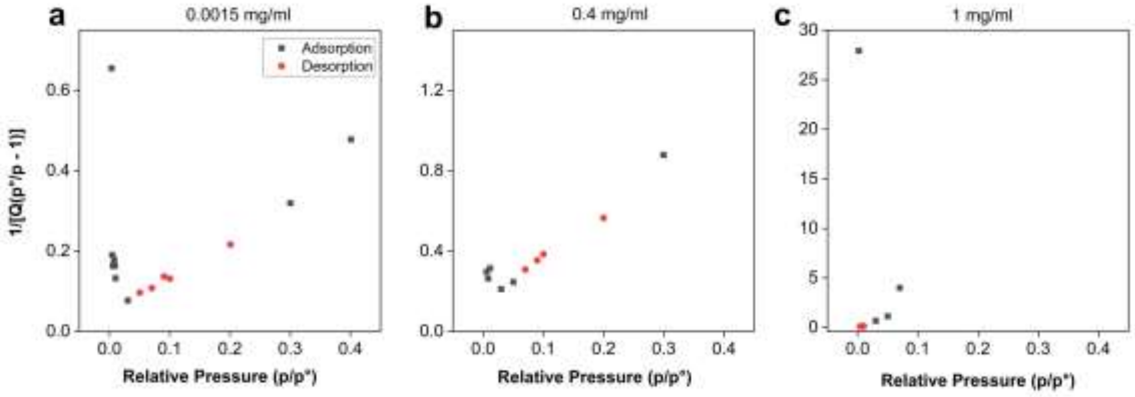

Figure S2. BET surface area analysis of BSA–trypsin sponges fabricated with increasing trypsin concentrations. BET plots based on nitrogen adsorption–desorption isotherms for sponges containing. (a) 0.0015 mg/mL, (b) 0.4 mg/mL, and (c) 1 mg/mL trypsin. The y-axis represents $1/[Q(P_0/P) - 1]$, where Q is the quantity of gas adsorbed at relative pressure P/P_0. At low trypsin concentration. (a), the linear trend suggests a moderately porous structure with measurable surface area. At 0.4 mg/mL (b), greater point dispersion indicates increased crosslinking and reduced pore uniformity. At 1 mg/mL (c), diminished signal and loss of linearity suggest significantly reduced surface area and pore accessibility due to dense matrix formation.

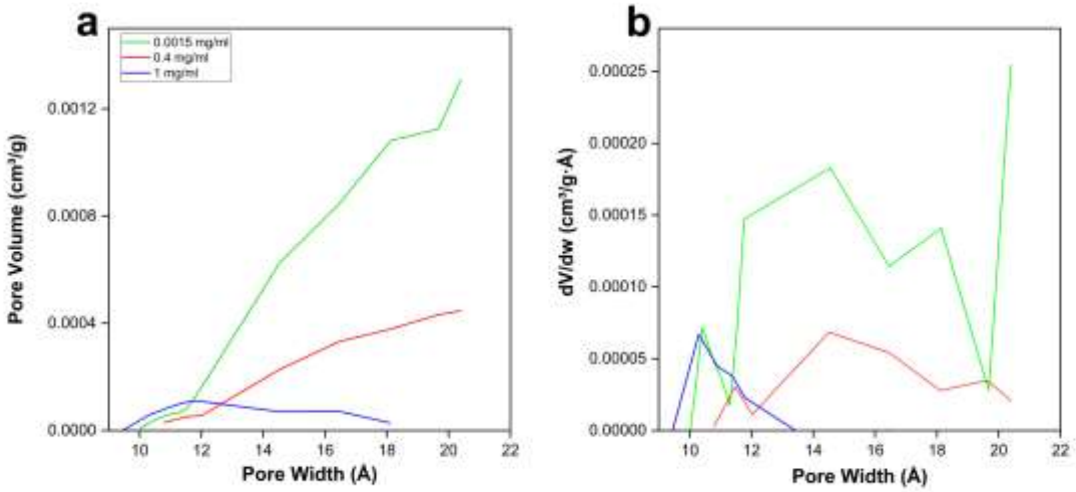

Figure S3. HK micropore analysis of BSA–trypsin sponges prepared with varying trypsin concentrations. (a) Pore volume as a function of pore width. Sponges containing 0.0015 mg/mL trypsin exhibit the highest micropore volume, indicating substantial micropore formation. At 0.4 mg/mL, the pore volume decreases with a modest peak, while sponges with 1 mg/mL show minimal microporosity, consistent with denser crosslinking. **(b)** Differential pore volume (dV/dW) versus pore width. The 0.0015 mg/mL formulation shows the most pronounced peaks, indicating abundant micropores.

Increasing trypsin concentration results in a stepwise reduction in peak intensity, reflecting decreased micropore density due to tighter network formation.

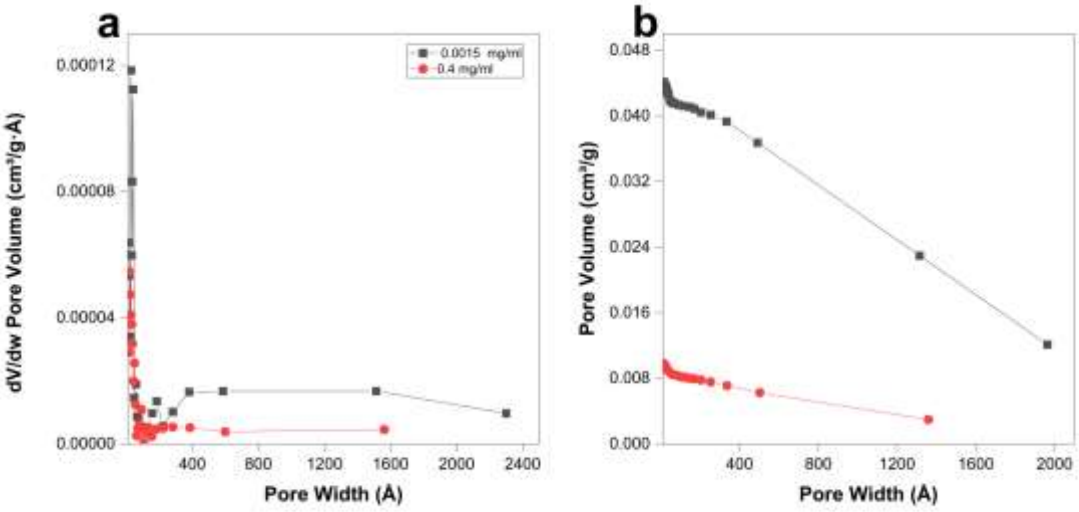

Figure S4. BJH mesopore analysis of BSA–trypsin sponges prepared with varying trypsin concentrations. (a) Differential pore volume (dV/dW) versus pore width. Sponges with 0.0015 mg/mL trypsin show higher mesopore density, particularly in the 0–200 Å range, reflecting reduced crosslinking and greater pore accessibility. At 0.4 mg/mL, dV/dW values are lower, indicating a reduction in mesopore formation due to increased network density. **(b)** Total pore volume versus pore width. The 0.0015 mg/mL sponges exhibit substantially greater pore volumes across the 0–400 Å range. In contrast, 0.4 mg/mL sponges show markedly reduced volumes, and mesopores were not detected in sponges fabricated with 1 mg/mL trypsin, consistent with extensive crosslinking and structural compaction.

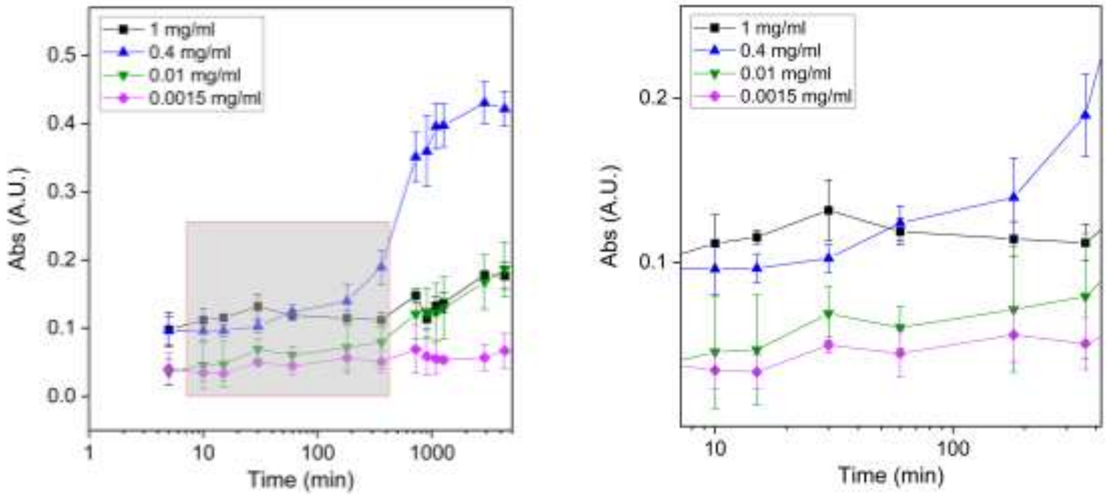

Figure S5. Kinetic Profiles of BAPNA Digestion by Sponges with Varying Trypsin Concentrations. Left: Full kinetic profiles of BAPNA cleavage by sponges containing different trypsin concentrations (0.0015–1 mg/mL), monitored by absorbance at 410 nm. Right: Zoomed-in view of the early kinetic phase (5–400 min) corresponding to the shaded region in the left panel. Sponges with 1 mg/mL trypsin exhibit an early plateau in product formation, suggesting rapid substrate depletion. In contrast, 0.4 mg/mL sponges display a more gradual and sustained increase in absorbance over the same time frame.

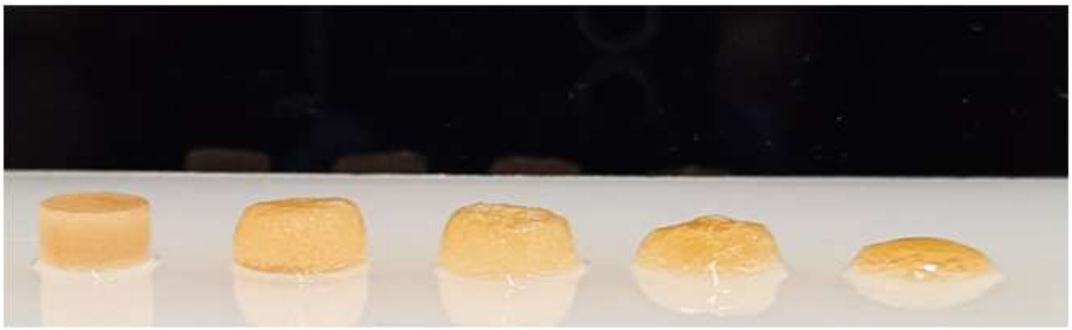

Figure S6. Visual comparison of BSA–trypsin sponges (0.4 mg/mL trypsin) prepared with increasing acetic acid (AA) concentrations: 0%, 0.1%, 0.5%, 0.75%, and 1.5% (left to right). AA modulates tyrosine–tyrosine cross-linking during photoactivation, thereby influencing sponge morphology and mechanical integrity. As AA concentration increases, sponges appear progressively

softer and less structurally rigid, reflecting reduced cross-link density. These morphological changes are consistent with enhanced flexibility and increased substrate diffusion capacity at moderate AA levels.

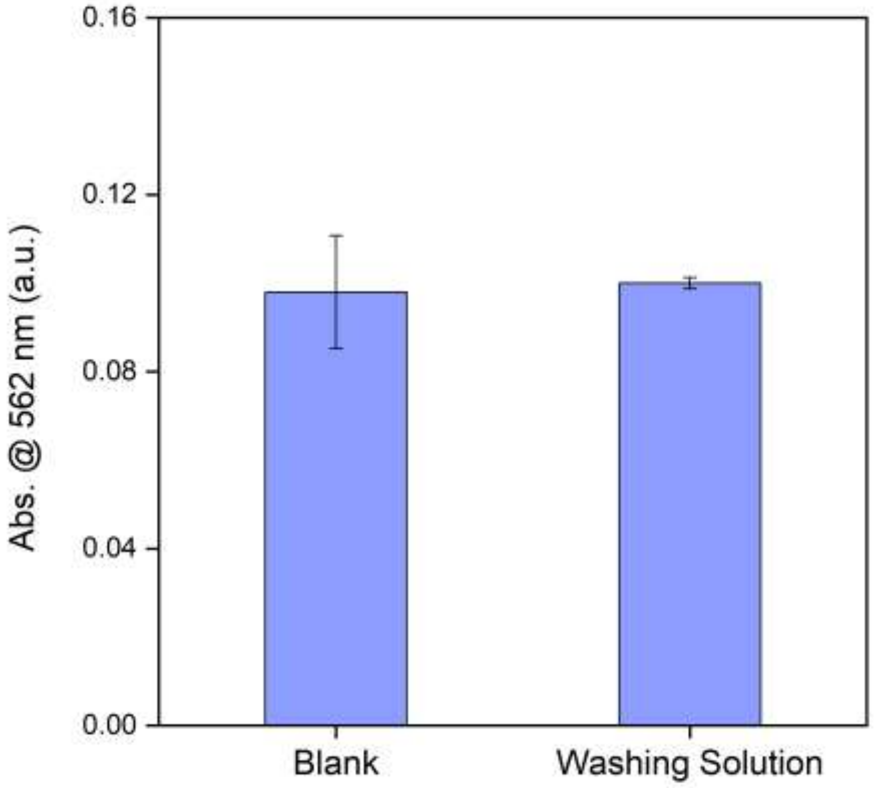

Figure S7. Assessment of protein leaching from BSA–trypsin sponges (1 mg/mL trypsin) using the BCA assay. Sponges were incubated in TRIS buffer over a 6-hour period, with buffer exchanged every 2 hours. At each interval, 25 μL aliquots were collected and analyzed via BCA protein assay (λ = 562 nm) to quantify potential protein release. Absorbance readings were comparable to the TRIS blank, indicating negligible protein leaching. These results confirm the structural stability of the sponge and the covalent retention of trypsin within the matrix.

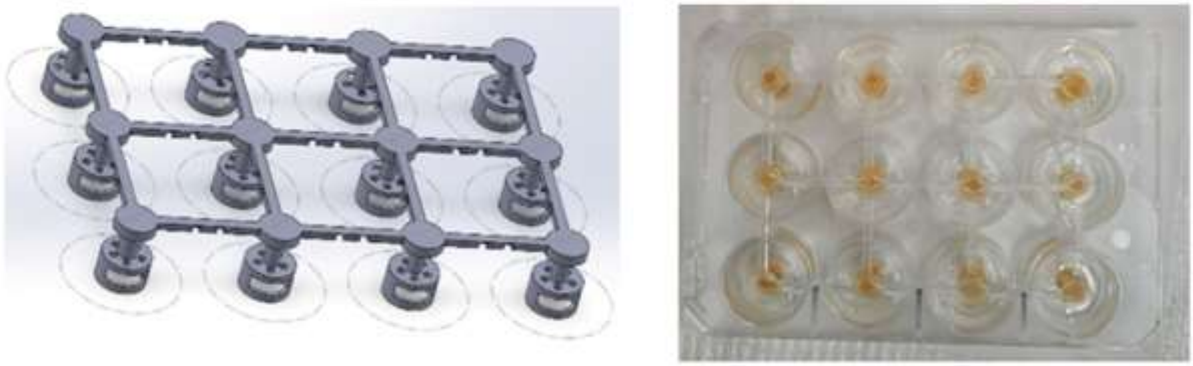

Figure S8. Custom scaffold system for standardized enzymatic activity assays in a 12-well plate. (a) CAD rendering of the 3D-printed scaffold designed to hold BSA–trypsin sponges during enzymatic assays. Each unit comprises a hollow cylindrical mold (9.5 mm diameter × 6 mm height) that fits securely into individual wells. The open-bottom design allows direct contact with substrate solution from below, while the top contains five perforations (1.5 mm diameter) to facilitate solution exchange from above. Side cutouts (7.6 mm × 2.75 mm) further enhance radial diffusion, ensuring full exposure of the sponge to the assay medium. **(b)** Photograph of the scaffold deployed in a 12-well plate containing sponges, demonstrating the practical assay setup for uniform and reproducible enzymatic activity measurements.
